# N-Butyl Cyanoacrylate Use for the Management of Type II Endoleak in Endovascular Aortic Repair: A Scoping Review

**DOI:** 10.3390/jcm15218596

**Published:** 2026-11-04

**Authors:** George Apostolidis, Petroula Nana, Giuseppe Panuccio, José I. Torrealba, Daour Yousef al Sarhan, Nils Zabel, Christian-Alexander Behrendt, Tilo Kölbel

**Affiliations:** 1 German Aortic Centre, Department of Vascular Medicine, University Medical Center Eppendorf, 20246 Hamburg, Germany; george.apostolidis4@outlook.com (G.A.); jitorrealba@gmail.com (J.I.T.);; 2 Department of Vascular and Endovascular Surgery, Asklepios Clinic Wandsbek, Asklepios Medical School, 20243 Hamburg, Germany; behrendt@hamburg.de

**Keywords:** N-Butyl-cyanoacrylate, NBCA, embolization, type II endoleak, endovascular aortic repair

## Abstract

**Objective:** Different endovascular techniques are available for type II endoleak management. This scoping review aimed to summarize the available evidence on the use of N-Butyl cyanoacrylate (NBCA) for type II endoleak management. **Methods:** This review followed the Preferred Reporting Items for Systematic Reviews and Meta-Analyses extension for Scoping Reviews (PRISMA-ScR). A protocol was registered on Open Science Framework. A systematic search using the PCC (Population, Concept, Context) framework was performed in PubMed, Scopus and Cochrane Library. Findings were systematically presented using structured tables summarizing NBCA techniques, efficacy and safety outcomes. **Results:** Following full-text assessment, 65 studies were included, comprising cohort and comparative studies, case reports, technical reports and preclinical studies. Single-arm cohort studies reported technical success rates of 90–100%, complication rates of 0–40% and variable durability outcomes, with clinical success ranging from 88 to 100%, endoleak resolution from 40 to 100% and aneurysm sac increase from 13 to 50%. Comparative studies reported improved outcomes with ethanol addition to the NBCA-ethiodized oil mixture compared with NBCA-ethiodized oil alone, shorter procedure times with direct puncture versus intra-arterial approach and more durable mid-term outcomes with selective embolization of contributing branches. Individually reported complications highlighted embolization risks and technical reports focused on catheter utilization. Pre-emptive embolization showed favorable outcomes on sac diameter reduction and endoleak type II occurrence. **Conclusions:** NBCA demonstrated technical feasibility for type II endoleak management, with variable durability. Considerable heterogeneity existed in access techniques, mixture ratios and outcome reporting. Preventive use demonstrated promising outcomes. Given the retrospective designs, limited sample sizes and heterogenous outcomes, adequately powered prospective studies are warranted.

## 1. Introduction

Despite low morbidity and mortality rates, endovascular aortic repair remains associated with procedure-specific adverse events requiring long-term surveillance, such as device migration, kinking or endoleaks [1,2,3]. Type II endoleaks, resulting from retrograde blood flow into the aneurysm sac through collateral aortic side branches, such as the inferior mesenteric artery or segmental arteries, are the most frequently encountered endoleak subtype after aortic repair [4]. Almost 15% of electively treated patients exhibited a type II endoleak at completion angiography in large registry cohorts [5,6]. Although most of them resolve spontaneously and are considered of benign nature, persistent type II endoleak may lead to aneurysm sac enlargement, potentially compromising the durability of the proximal seal, increasing aneurysm pressurization and risk of rupture, as well as the need for reinterventions. Pre-emptive embolization of such side-branches has been proposed as an effective measure in well-selected patients, but its value remains controversial [4,7,8].

Various embolization techniques have emerged for the management of type II endoleak, including transarterial, translumbar, transcaval, or combined, selective or non-selective, approaches [9]. Embolic agents include solid materials, like coils and plugs, and liquid agents, such as thrombin, fibrin glue, ethylene-vinyl alcohol copolymer (ENOH) and N-butyl-cyanoacrylate (NBCA) mixtures [9]. NBCA has emerged as a compelling embolic alternative, either alone or in combination with adjunct materials, due to its favorable characteristics, including firm occlusion, coagulation independency, as well as adjustable viscosity and enhanced radiopacity, based on the ratios of its mixture with ethiodized oil [10]. Available evidence on the use of NBCA for type II endoleak embolization indicates high technical success with variable long-term clinical success [11,12]. Although the safety profile appears acceptable, significant adverse events have been described [13,14].

This scoping review aimed to systematically summarize the currently available literature on the use of NBCA for the management of type II endoleak in patients undergoing endovascular aortic repair, focusing on technical approaches, efficacy and safety outcomes.

## 2. Methods

### 2.1. Study Design and Eligibility Criteria

A scoping review of available literature was planned and performed according to the Preferred Reporting Items for Systematic Reviews and Meta-Analyses extension for Scoping Reviews (PRISMA-ScR) [15]. A pre-defined protocol was published in the Open Science Framework (OSF) registry (https://osf.io/98ejm; accessed on 7 July 2026). No approval by an ethics committee was required due to the study design.

Available literature, including observational studies (prospective and retrospective), interventional studies, case reports and technical notes that were published in English up to 25 June 2026 were eligible. Studies were eligible if they reported on patients undergoing endovascular aortic repair of any extent who received NBCA embolization either preemptively (during or before the index procedure to prevent type II endoleak) or therapeutically (after the index procedure for the treatment of an established type II endoleak). No restrictions were applied regarding the endograft design, the arteries related to type II endoleak, or the access approach. In vitro studies or studies performed on animal subjects were also included. Adjunctive use of other materials along with NBCA was considered acceptable.

Studies reporting on other types of endoleaks (e.g., Ia or gutter endoleaks) or the use of NBCA for interventions other than type II endoleak (preventive or therapeutic) management were excluded. Studies reporting mixed populations managed with different liquid embolic agents were excluded when NBCA-specific outcomes could not be extracted separately.

### 2.2. Information Sources and Search Strategy

A systematic literature search of English articles in PubMed, Cochrane Library and Scopus was conducted. This search was planned and performed based on the PCC (Population, Concept, Context) framework (Supplementary Table S1). The following terms, including Expanding Medical Subject Headings (MeSH Terms), were used: “endoleak”, “aneurysm sac perfusion”, “NBCA”, “n-butyl-cyanoacrylate”, “cyanoacrylate”, “Glubran”, “Histoacryl”, “Trufill”, and “glue” (Supplementary Table S2). The reference lists of individual eligible studies were screened for eligible articles, as a measure to account for the gray literature. The literature search was last updated on 25 June 2026, across all three online databases.

The retrieved literature was independently reviewed by two authors (G.A. and P.N.), in both stages of title and abstract screening, as well as full text assessment. Any disagreement was resolved after discussion with a third author (T.K.).

### 2.3. Data Collection

The data collection was performed using a standardized Excel file (Microsoft Corp., Redmond, WA, USA) that was created, and pilot tested by two authors (G.A. and P.N.). Collected data included, when available:

- Study baseline characteristics (author, year, journal of publication, study design, study period, eligibility criteria, objective);
- Baseline information regarding patients (sample size, age, sex, underlying disease, setting of repair, type of endovascular aortic repair, follow-up time, anticoagulation status, comorbidities);
- Endoleak characteristics (feeding vessels, diagnostic imaging);
- Technical details (type of approach, material details, NBCA mixture composition, use of adjuncts, outcome definitions);
- Outcomes (reported clinical and technical outcomes, including effectiveness and complications, as available in the included studies).

The data collection process was performed independently by two authors (G.A. and P.N.). Any disagreements were resolved after thorough discussion with a third author (T.K.) until consensus was reached.

### 2.4. Quality Assessment

A formal critical appraisal of the methodological quality or risk of bias of the included studies was not performed. Although planned in the protocol if deemed appropriate, it was ultimately considered beyond the scope of this scoping review, given the descriptive nature of the available evidence, which included diverse study designs and heterogenous outcome reporting, further limiting the comparability of the evidence.

### 2.5. Synthesis and Presentation of the Results

A descriptive and narrative synthesis was performed. Findings were summarized according to study characteristics, technical approaches and reported outcomes. Due to substantial heterogeneity in study designs, patient populations, and reported outcomes, no quantitative synthesis was conducted.

Results were organized to map patterns of NBCA use, including technical strategies, efficacy outcomes and safety profiles in the existing literature. Respective summary tables were created. Numerical data were presented as counts and percentages for categorical variables and either mean ± standard deviation or median with range/interquartile range, when available or appropriate.

## 3. Results

### 3.1. Study Selection

**Figure 1:**
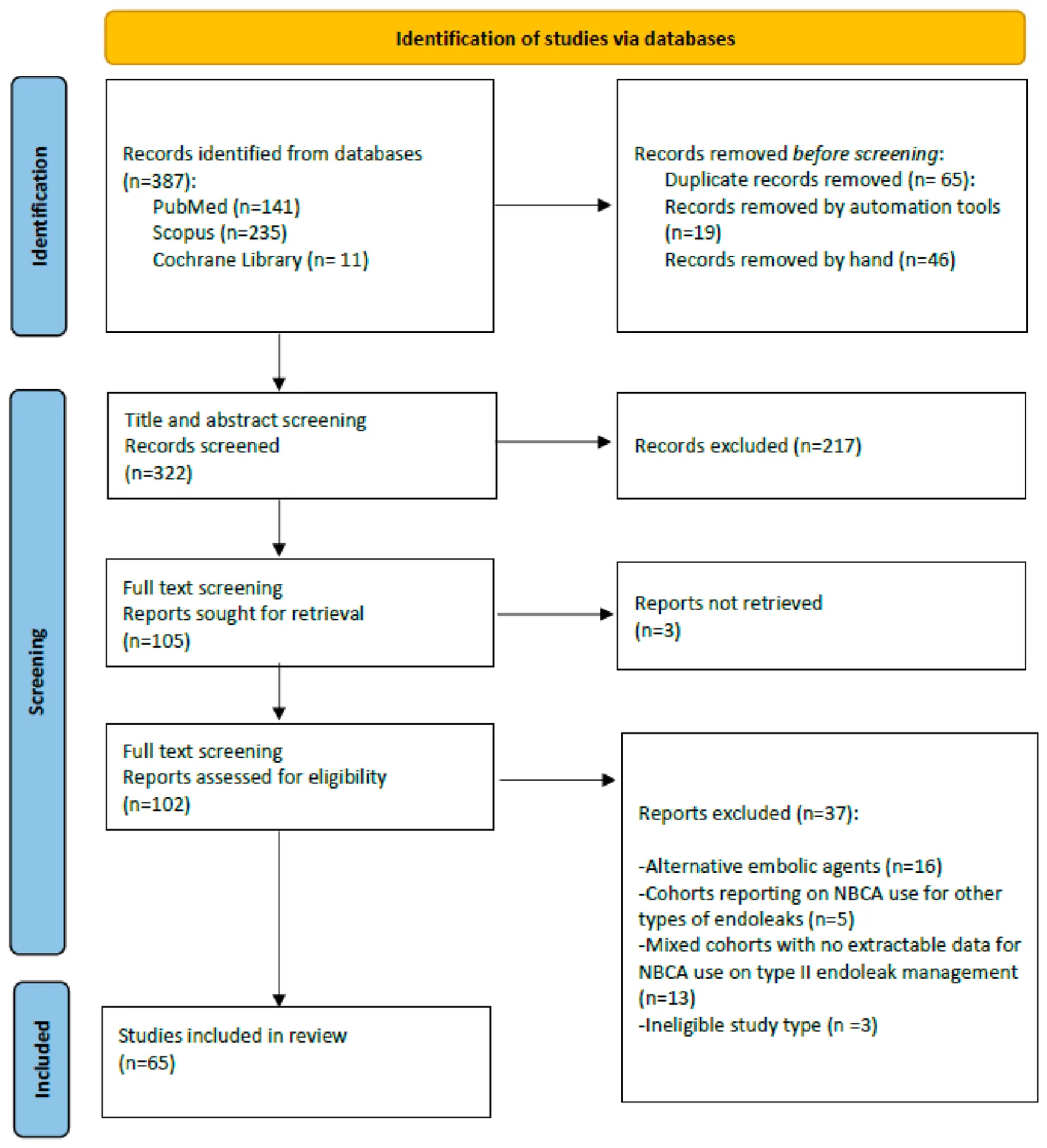
PRISMA flow diagram of the scoping review.

The systematic search of the literature initially identified 387 study reports. After the deduplication process and eligibility evaluation, 65 studies were included in this scoping review (Figure 1).

Among the included studies, four were non-human experimental studies, 16 were observational cohort studies, comprising 13 single-arm and three comparative studies, and 29 were case reports, including 13 reports describing the use of NBCA and 16 reports focusing on significant complications. Moreover, 11 studies were technical reports, analyzing various aspects, one study evaluated the cost-effectiveness of two NBCA alternatives and four studies focused on pre-emptive embolization outcomes. Across the included human studies comprised a cumulative sample size of 607 NBCA-treated patients was reported, although potential overlap between patient populations across different publications could not be excluded.

### 3.2. Non-Human Studies

NBCA was first experimentally used for type II endoleak management in swine models in 2000 by Schurink et al., who investigated the effect of NBCA on endoleak exclusion, aneurysm sac pressure and thrombosis [16]. On the experimental basis of a swine model, Rolland et al. have also shown that the pre-emptive injection of an NBCA mixture (NBCA with ethiodized oil at 1:4 ratio) into the aneurysm sac and feeding arteries can prevent endoleak formation and persistence after endovascular aortic repair while triggering a restenosis-related tissue reaction of the aortic aneurysm [17]. In 2018, another study on swine models used an NBCA–ethiodized oil–ethanol mixture (1:5:1 ratio) and showed successful endoleak type II management in all four cases immediately after the embolization, as well as aneurysm sac thrombosis, which persisted three months after the intervention [18]. According to the authors, the addition of ethanol to the mixture may enhance the safety due to its decreased adhesion to the catheter wall and less damage to the aortic tissue, preserving the efficacy of the technique [18].

An in vitro study by Barge et al., using a flowing model simulating an aneurysm sac with endoleak, was used to assess the catheter adhesion while administering NBCA through translumbar access [19]. Although no resistance was noted for metallic needles and straight catheters, curved catheters and sheaths adhered to the aneurysm sac [19].

### 3.3. Single-Arm Cohort Studies

Of 13 single-arm single-center cohort studies (including case series) that were eligible for this review, Charitable et al. presented the largest cohort among eligible studies, consisting of 30 patients treated via a translumbar approach for type II endoleak after EVAR [20]. Seven studies reported on mixed cohorts including either alternative endoleak types or alternative embolic agents; however, results on NBCA use for type II endoleaks were extractable [12,21,22,23,24,25,26]. Voigt et al., although including 32 cases, reported on prevalence of periaortic inflammation in this cohort (4/32) and focused on analyzing outcomes of these patients [26]. A transcaval approach was only reported in one study [27]. Seven studies analyzed outcomes of small series, including less than ten patients [22,23,24,25,28,29,30]. One study, including three NBCA-embolized patients, reported, additionally, on the cost-effectiveness of a watchful strategy, leading to reintervention for type II endoleak after six months and as long as an aneurysm sac increase is present [23]. Chao et al. systematically performed coil embolization of IMA before NBCA application to avoid embolization complications [30], while Nakai et al. aimed to assess the efficacy of Technetium- 99m SPECT in follow-up imaging assessment of embolized patients [25].

In total, 176 patients were treated via an intra-arterial (66 cases), translumbar (95 cases), transabdominal (seven cases) or transcaval access (10 cases). Although the outcomes of interest were heterogenous, technical success was evaluated in six studies and ranged between 90 and 100% [12,21,22,24,25,28], with endoleak resolution (seven studies) ranging between 40 and 100% [11,20,22,25,28,29,30]. Endoleak recurrence was evaluated in three studies and ranged between 0 and 30% [21,23,27]. The clinical success was evaluated in three studies and ranged between 88 and 100% [12,22,24], while reinterventions due to reoccurrence (four studies) ranged between 12 and 15% [11,20,28,30], and aneurysm sac increase (four studies), ranging between 13 and 50% [20,22,27,29].

Among these studies, any type of complication was reported in nine studies [11,20,22,23,24,26,27,28,29,30], and ranged between 0 and 40%, with six studies reporting a complication rate of 0% [20,23,24,28,29,30]. The type of complication was post-operative back pain in four patients [22,27], NBCA muscle infiltration leading to back pain and fever in two patients [11], and a case of a vena cava minor extravasation [27]; all reported complications were self-limiting. One study reported only on periaortic inflammation after NBCA application [26]. The NBCA mixture consisted of NBCA and ethiodized oil at variable ratios among studies, ranging between 1:1 and 1:10, and it was used along with coils in some cases. Median/mean follow-up ranged from 6 months to 83 months (Table 1).

### 3.4. Comparative Studies

Three single-center studies provided extractable outcomes on management of type II endoleak using NBCA embolization (Table 2). The respective studies focused on comparing different mixture compositions [31] and access routes [32,33].

Comparison between patients treated with an NBCA–ethiodized oil (NE) mixture versus an NBCA-ethiodized oil–ethanol (NEE) mixture, revealed significant superiority of the NEE in freedom from sac enlargement and freedom of reintervention at one year. No adverse events were reported, while the access route was either intra-arterial or through direct sac puncture (translumbar or transabdominal), differences between groups in a subgroup analysis for direct sac access-managed patients remained statistically significant [31].

In an early study from 2009, intra-arterial type II endoleak management using coils and translumbar management using coils and NE mixture were compared demonstrating no significant difference in terms of technical, clinical success and major complications. However, translumbar access was related to lower clinical success (72.6% vs. 78.3%) and two major complications, including bowel ischemia of embolic nature and retroperitoneal bleeding [32]. A later study comparing direct sac puncture embolization with NE to intra-arterial embolization with NE, with or without adjunctive coil deployment, showed similar technical success but a significant difference in procedure time and contrast use, favoring direct sac puncture [33].

### 3.5. Case Reports

Multiple case reports have reported on patients managed with NBCA for type II endoleaks (Table 3). The first (chronological) report demonstrates the adjunctive role of NBCA mixture in the successful management of a recurrent type II endoleak after endovascular abdominal aortic aneurysm repair (EVAR) [34]. Subsequent reports confirm further the efficacy of this method in emergent setting, where either ruptured abdominal aortic aneurysms (AAAs) or aortocaval fistulae were treated [35,36,37,38]. Cases of co-existent multiple endoleaks are also reported, including one report of transgraft embolization with subsequent endograft relining [35,36,39].

NBCA embolization was also applied after arch repair with FET, due to an LSA-related type II endoleak, while type II endoleaks after iliac aneurysm repair were also reported [40,41,42]. Special access routes, including transgluteal or complex intra-arterial access (due to previous hybrid repair) have been documented [42,43]. Uncommon imaging modalities, including carbon-dioxide angiography, photon emission computer tomography and non-contrast magnetic resonance tomography, were used to assist intraoperative technical success or post-operative follow-up [44,45,46]. The existence of an extended vascular network in the aortic wall (vasa vasorum), feeding the endoleak nidus, was, repeatedly, noted [40,41,44].

**Table 3:** Case reports presenting cases managed with NBCA for type II endoleak.

Author	Year	Clinical Context	Access Route	NBCA Mixture	Follow-Up	Special Features
Wilmot et al. [34]	2007	Recurrent type II EL after EVAR	TL-nS	NE (1:2)	-	Previous IMA endoleak treated with coils, concurrent LA endoleak treated with coils
Fukunishi et al. [44]	2026	Vasa vasorum (Moyamoya) type II EL after EVAR	IA-S	NE (1:4)	1 year	CO_2_ angiography to reduce antegrade flow and avoid embolization distally
Hamada et al. [41]	2019	Type II EL from reticular artery after EVAR for CIAA	IA-S	NE (1:5)	9 months	Endoleak from reticular artery arising from DCIA and being anastomosed to a network of reticular arteries in the aortic wall
Claudio et al. [42]	2023	Type II EL from iliolumbar artery after exclusion of an IIAA	TG-nS	-	1 month	Endoleak despite Onyx sac embolization, initial transarterial attempt failed, transgluteal succeeded
Chiba et al. [40]	2026	Type II EL from atypical mediastinal collateral artery	IA-S	NE (14.5% NBCA)	1 year	Previous FET with extra-anatomic LSA bypass, endoleak from atypical mediastinal branch connected to a network of vasa vasorum
Onishi et al. [43]	2023	Type II EL from CT after hybrid repair of TAA	IA-S	NE (1:5)	8 days	Branches of CT initially coil embolized- endoleak from dorsal pancreatic artery- selectively embolized through bypass graft and SMA
Nakai et al. [39]	2019	Mixed endoleak (I, II and III) after EVAR	TGr-nS	NE (1:4)	6 months	Transgraft embolization, concurrent proximal and distal extension, kissing endograft, main endograft relining
Kuziez et al. [35]	2018	Ruptured AAA due to mixed EL (type II from LAs and Ia) after EVAR	TL-nS	NE (1:2.5)	18 months	Emergent setting, concurrent proximal extension with snorkel for LRA
Laganà et al. [36]	2008	Ruptured AAA due to mixed EL (type II from iliolumbar and IIIa) after EVAR	IA-S	NBCA	6 months	Emergent setting, concurrent type IIIa endoleak from left limb disconnection
Maleux et al. [37]	2025	Ruptured AAA due to type II EL from LAs after EVAR	TL-nS	NE (1:3)	1 year	Emergent setting
Burke et al. [38]	2003	Aortocaval fistula supplied by type II EL from IMA	IA-S	NE (1:3)	1 year	Aortocaval fistula, emergent setting
Pietura et al. [45]	2022	Type II EL from iliolumbar arteries after EVAR	IA-S	NE (16% NBCA)	Residual endoleak post-op	NCE-MRI for the assessment of result
Nakai et al. [46]	2014	Type II EL after EVAR	TA-nS	NE (1:2) + coils	12 months	Use of Technetium-99m HSAD-SPECT

Footnote: EL: endoleak; EVAR: endovascular aortic repair; LA: lumbar artery; CT: celiac trunk; IMA: inferior mesenteric artery; CIAA: common iliac artery aneurysm; IIAA: internal iliac artery aneurysm; TL: translumbar; IA: intra-arterial; TG: transgluteal; TGr: transgraft; nS: non-selective; S: selective; NBCA: n-butyl cyanoacrylate; NE: mixture containing n-butyl cyanoacrylate and ethiodized oil; CO_2_: carbon dioxide; FET: frozen elephant trunk; LSA: left subclavian artery; NCE-MRI: non-contrast enhanced magnetic resonance imaging; DCIA: deep circumflex iliac artery; HSAD-SPECT: human serum albumin diethylenetriamine pentaacetic acid single-photon emission computer tomography.

### 3.6. Technical Studies

Eleven technical studies were included, focusing mainly on catheter systems (Table 4). Forty-one cases were analyzed, with the intra-arterial approach being the most commonly use (7/11 studies). Five studies aimed to present multi-coaxial systems of catheters and microcatheters, used to achieve effective embolization via an intra-arterial access [47,48,49,50,51], while one study described the applied coaxial catheter system for a direct-puncture technique [52]. The use of flow-directed microcatheters has been supported as an effective alternative [53]. Balloon occlusion of the aorta during the embolization process was, also, described as a means to enhance efficacy and safety of embolization [51,54]. Moreover, the use of a transeptal needle-sheath to establish a transcaval access and the use of isocenter marking and contrast-enhanced ultrasonography for the application of transpendicular and transabdominal access have been described [55,56,57]. Different mixture ratios were used, ranging from 1:2 to 1:5; The respective information and clinical outcomes, are presented in Table 4.

### 3.7. Complications

Case reports have been published focusing on clinically significant or unusual complications resulting from interventions aiming to manage type II endoleaks with the use of NBCA (Table 5). Nine of them include complications of embolic cause [14,58,59,60,61,62,63,64,65]. Colonic ischemia with subsequent need for colectomy or conservatively managed ischemic colitis have been reported after IMA embolization [60,63,64], while management of endoleaks involving lumbar arteries has led to neurological adverse events of variable severity involving lower limb motor function, with only one out of four demonstrating complete resolution [14,58,59,61]. NBCA lumbar artery embolization has also been reported to cause skin ulcerations, resulting due to muscle infiltration of the embolic substance [65]. Distal glue embolization has been recorded, with subsequent need for peripheral lower limb revascularization [62].

Acute limb ischemia due to iliac limb external compression caused by the embolic agent in four cases [13,66,67,68]. Three of them were treated acutely due to symptomatic state with kissing balloon angioplasty [13,66,68], while one asymptomatic patient was conservatively managed [67]. Aortic graft and/or aortic infection has also complicated such interventions, leading in all cases to extensive open aortic reconstructions [65,69,70,71].

### 3.8. Cost-Effectiveness

A cost-effectiveness study comparing two alternative commercial preparations, Trufill and Histoacryl, was published in 2021. After comparing 20 Trufill to 14 Histoacryl, managed in a single tertiary center in the USA, between March 2011 and September 2018, for type II endoleak cases, managed via translumbar approach, results have shown that the procedure cost was significantly higher for Trufill, with more coils and less glue used in this group. Percentage change in sac diameter did not differ between groups and no complication was reported [72].

### 3.9. Pre-Emptive Embolization

Pre-emptive embolization using NBCA was reported by four single-center studies. Selective and non-selective pre-emptive embolization of aortic side branches (lumbar arteries and inferior mesenteric arteries) with NBCA, as a measure to prevent type II endoleak during endovascular aortic repair of AAA demonstrated a 3.2% of aneurysm sac diameter reduction and 17% persistent endoleak type II after 6 months [73]. Nakai et al. applied non-selective pre-emptive embolization using NEE mixture in 24 patients with endovascular repair of AAA, with either infrarenal neck angulation of >60° or AAA diameter of >60 mm, and reported low systolic sac pressure indexes (systolic pressure in aneurysm sac/systematic systolic pressure), no aneurysm diameter increase and a 12.5% persistent endoleak type II rate at 12 months [74].

A comparative study between patients treated with EVAR alone versus EVAR combined with non-selective NE pre-emptive embolization demonstrated significant superiority for the embolization group regarding type II endoleak occurrence at 7 days and AAA diameter reduction at 12 months [75]. An updated version of this study confirmed these findings and reported a significant difference in survival without AAA enlargement and survival without reintervention, favoring the embolization group (Table 6) [76].

Beyond its therapeutic application for established type II endoleaks, NBCA embolization has also been investigated in a limited number of studies as a pre-emptive strategy during EVAR. Although these approaches represent distinct clinical strategies, both ultimately aim to enhance the durability of endovascular aortic repair by eliminating sources of persistent aneurysm sac perfusion, thereby reducing the risk of subsequent sac enlargement and related complications.

## 4. Discussion

This scoping review systematically mapped the available evidence regarding the use of NBCA for the management of type II endoleak in patients receiving endovascular aortic repair. Non-human studies proved feasibility of both therapeutic and preventive application of the technique [16,17,18,19]. Available cohort studies including multiple alternative approaches and NBCA mixture ratios, indicated high rates of technical success and variable rates of clinical success, assessed through endoleak resolution and recurrence, aneurysm sac size changes and reinterventions [11,12,20,21,22,23,24,25,26,27,28,29,30,31,32,33]. Reported complications were uncommon, mainly reported in case reports, and comprising both embolic and non-embolic complications, such as endograft infection and compression; potential underreporting should be considered, further compounded by the limited availability of long-term follow-up data [13,14,58,59,60,61,62,63,64,65,66,67,68,69,70,71]. Technical reports assessed the performance of multiple alternative catheter systems and access approaches, demonstrating acceptable results [47,48,49,50,51,52,53,54,55,56,57]. Use of NBCA for the prevention of type II endoleak is also feasible with available studies reporting high outcome durability and low complication rates [73,74,75,76].

Available guidelines suggested endoleak type II management, based on aneurysm sac growth criteria, but no specific method or technique was proposed [2]. Compared to previous cumulative evidence on type II endoleak management with various embolic agents, suggesting highly variable technical success and modest clinical success rates, the included studies suggested generally favorable technical success with NBCA embolization, while conclusions regarding its long-term effectiveness and durability remained limited by the heterogenous methodological characteristics of the available studies [9,77]. Aneurysm sac increase rates and reintervention rates were in agreement with previous cumulative evidence [77,78]. With regard to pre-emptive embolization with NBCA or other material, the available evidence was still controversial regarding the benefits of such an approach, with selected cases benefiting from lower endoleak type II occurrence, reintervention and sac increase [75,76,79]. The importance of durable type II endoleak management is further highlighted by the potential consequences of persistent sac perfusion. Although type II endoleaks are not identified as the primary cause of EVAR failure and aneurysm rupture, persistent sac perfusion may contribute to aneurysm sac enlargement and subsequent aortic morphological changes, potentially compromising the proximal sealing zone and promoting secondary type I (or even III endoleaks). Such complications may increase the risk of rupture and necessitate complex reinterventions, including endograft explantation, which are associated with substantial morbidity and mortality. However, the impact of specific embolization strategies, including NBCA, on preventing such outcomes remains unclear [80,81].

NBCA mixtures are mainly built from NBCA plus an oil-based contrasted agent, ethiodized oil (Lipiodol; Guerbet, Villepinte, France). This additional agent is mixed with NBCA in variable ratios and acts like a radiopaque carrier and diluter, modifying its polymerization time [82]. High ethiodized oil content is preferred for non-selective embolization, while mixtures containing less ethiodized oil are chosen for selective embolization, to induce faster polymerization and avoid distal embolism of the mixture [73]. Although this constitutes the general rule, it is not always strictly followed and ratios are not standardized but are rather operator-dependent, as is confirmed in this review [83]. Several commercially available NBCA-based formulations exist, including Histoacryl (B. Braun, Melsungen, Germany), Glubran 2 (GEM S.r.l., Viareggio, Italy) and Trufill (Cerenovus, Irvine, CA, USA), which differ in composition, additives and polymerization characteristics. Although these preparations have been used for embolization procedures, comparative evidence regarding their use in type II endoleak management remains limited, and current practice appears mainly driven by operator preference, availability and local regulations [72,84].

The addition of ethanol to the mixture was proposed, indicating its positive effect on better handling of the mixture, lower risk of adhesion to the catheter and a better profile of vascular wall changes [85]. Available evidence on type II management, supports the superior outcomes of the mixture [31]. Mixture temperature also plays a role in its viscosity with evidence supporting higher temperature leading to reduced viscosity [86]. The addition of coils to the strategy was also described multiple times, but no available evidence reported any difference between these alternatives while the application of coils for selective embolization of specific feeding vessels to securely prevent NBCA mixture embolization seems reasonable [30]. Conversely, the use of liquid embolic agents may be particularly advantageous in cases involving complex networks of type II endoleak feeding vessels (vasa vasorum), with individual reports supporting the feasibility of NBCA embolization in these settings [40,44].

The access route for the application of this embolization with NBCA also showed great heterogeneity across the included studies. Intra-arterial and translumbar accesses were the most commonly used, providing similar efficacy and safety outcomes, with translumbar puncture being superior in terms of procedural time and contrast volume [32,33]. The transabdominal approach was also described under appropriate imaging guidance [33,57]. Transcaval embolization also constituted a concrete alternative presenting excellent technical success and almost 90% of clinical success and freedom from type II endoleak recurrence [78]. Alternative unusual access routes, like transgluteal, transpedicular or transgraft were used, proving the customizability of the technique, but reducing the standardization of the methodology [39,42,56]. Both selective and non-selective embolization can be applied, with low-powered comparative evidence showing significantly higher clinical success for the selective one [87].

Available studies reported comparable or potentially favorable outcomes with NBCA relative to previously reported evidence on other embolic materials, although the strength of these findings remains limited by the methodological characteristics of available evidence. In a cohort where multiple embolic agents were applied, including NBCA in combination with coils, coil embolization alone was associated with a higher rate of reinterventions while another cohort study demonstrated significantly higher technical success with NBCA compared to other liquid embolic agents and coils [12,88]. Sac enlargement was not shown to be affected by the choice of coil alone or NBCA, with or without coils [89]. Similarly to NBCA, the alternative liquid embolic agent ethylene-vinyl-alcohol copolymer (Onyx; Medtronic, Irvine, CA, USA) demonstrated high technical success and variable clinical success [90,91]. Fibrin glue also showed enhanced effectiveness and durability, particularly in the setting of preventive embolization, as supported by high quality studies [92,93].

Despite generally favorable outcomes reported in cohort studies, NBCA mixture properties and the lack of adequate technique standardization bear an inherent risk of embolic complications [11,20]. The adhesive properties of the mixture may complicate the procedure, with adhesion of the agent to the catheter and may lead to endovascular foreign body entrapment [19]. Additionally, the initial fluidity of the embolic agent can lead to unplanned polymerization in an undesired vascular territory, including the aneurysmal sac and iliac arteries, and subsequent endograft compression [66]. Aortic graft infection is a rare complication and may be related to the need for multiple reinterventions, although robust evidence is lacking [94]. The interpretation of complication rates remains limited due to the heterogenous reporting, small study populations, and inconsistent follow-up across the available literature.

Currently available evidence includes several studies reporting mixed populations in terms of endoleak type and embolic agent, limiting the extraction of NBCA-specific outcomes and robust conclusions regarding its performance. Comparative studies involving predominantly NBCA-managed patients evaluated selective versus non-selective embolization and direct puncture versus intra-arterial approaches, reporting shorter intraoperative times and reduced fluoroscopy with non-selective technique and direct puncture [95,96]. Cohort studies reported favorable efficacy and safety outcomes with both intra-arterial and transabdominal approach, while one study assessing long-term durability of the NBCA embolization outcomes highlighted the low five-year freedom of aneurysm sac increase [88,97,98].

## 5. Limitations

The available evidence is predominantly based on retrospective, underpowered cohort studies and case reports, limiting the overall strength of conclusions. Substantial heterogeneity exists across studies regarding access approaches, embolization techniques, feeding vessel selectivity, NBCA mixture ratios and use of adjunctive embolic materials. Added to this, confounding by indication is inherent in the included single-center, non-randomized, retrospective studies with the above-mentioned heterogenous characteristics, as treatment selection may have been influenced by anatomical characteristics, operator preferences and institutional practices, limiting causal interpretation of the reported differences, including those reaching statistical significance (*p* < 0.05). Follow-up duration and outcome reporting were also inconsistent, reducing the comparability between studies. The reported outcomes, particularly complications, may be influenced by reporting bias. Heterogeneity in commercially available NBCA formulations may represent an additional confounder, potentially affecting technical performance and reported outcomes. Comparative analyses’ availability was scarce. Furthermore, overlap of patient populations across studies from the same institutions is highly likely, although its extent could not be determined. Consequently, the cumulative sample size may not represent the number of individual included patients.

Although formal quality assessment was not performed, consistent with the descriptive objectives of this scoping review, the absence of such an assessment limits the strength of conclusions regarding the efficacy and safety of NBCA embolization. Additionally, the restriction to English-language publications may have resulted in the omission of relevant evidence, particularly considering the substantial contribution of research centers from non-English speaking countries to the available literature. Future adequately powered, prospective studies, with systematic outcome assessment, addressing specific technical aspects like feeding vessel selectivity, access routes and NBCA mixture composition are necessary to enhance the quality of the available evidence and assist the standardization of NBCA embolization application.

## 6. Conclusions

Available evidence suggests that NBCA embolization for type II endoleak management is technically feasible, with generally favorable reported safety but variable durability. Considerable heterogeneity exists between the available studies, in terms of access approach, NBCA mixture ratios, adjunctive use of other embolic materials and reported outcomes.

A limited number of studies reporting on pre-emptive embolization using NBCA has shown promising short-term endoleak type II occurrence and aneurysm sac increase with an acceptable safety profile. The heterogenous and underpowered existing literature for both management and prevention of type II endoleak using NBCA prompts future studies of higher quality.

## Figures and Tables

**Table 1:** Cohort studies (including case series) reporting on cases managed with NBCA embolization for type II endoleak.

Author	Year	Sample	Access Route	NBCA Mixture	Main Findings	Follow-Up
Lagios et al. [11]	2018	25	TL-S	NE (23–50% NBCA)	- Endoleak resolution (one-day) 88%; - Reintervention 12% (same patients); - Two complications: NBCA infiltration of the psoas muscle leading to back pain and fever.	18.3 ± 7.3 months
Jiang et al. [21] *	2025	25	IA-S	NE (1:8–10)	- Technical success 100%; - No endoleak recurrence; - Non-AAA-related deaths (during follow-up) 8%.	Not reported
Hamidizadeh et al. [12] *	2021	20	IA-S: 9, TL-nS: 11	NE (1:1–1:3) ± coils	- Technical success 90%; - Clinical success 88%; - NBCA significantly higher technical success compared to coils, Onyx ± coils, coils + gelfoam.	44.7 ± 33.5 months (whole cohort)
Charitable et al. [20]	2019	30	TL-nS	NE (1:2.5) ± coils	- Immediate endoleak resolution 50%; - Eventual sac stabilization 36.7%; - Reintervention for persistent type II endoleak 13.3%; - Larger aneurysm diameter + antiplatelets: significantly favored persistent endoleak; - No complications.	19.1 (95% CI 11.1–27.2) months
Voigt et al. [26] *	2026	32	IA: 20 TL: 12	NE ± coils or ENOH	- Periaortic inflammation 4/32 (12.5%); - Three antibiotics and one EVAR explantation + axillobifemoral bypass; - No difference in periaortic inflammation between embolics.	Not reported
Van Sickler et al. [27]	2023	10	TC-nS: 10	NE (1:2–10)	- Endoleak type II on follow-up 30%; - Sac expansion > 5 mm 30%; - Complications: one vena cava self-limiting extravasation, two back pains.	6.5 (0–14.8) months
Kajiwara et al. [22] *	2016	5	IA: 4-S TA: 1-nS	NE (1:2–10) + coils	- Technical success 100%; - Clinical success 100%; - Endoleak resolution 40%; - Aneurysm sac increase 20%; - Two complicated with back pain (one had additional self-limiting let-leg muscle weakness).	6± 6.2 months
Stavropoulos et al. [28]	2005	9	TL-nS	NE (1:2)	- Technical success 100%; - Endoleak resolution 66%; - Reintervention 22%; - No complications.	83 (32–179) months
Steinmetz et al. [23] *	2004	3	TL-nS	NE (1:2–10) ± coils	- No endoleak recurrence; - No complications; - Survival at 48 months 78%.	16 (6–26) months
Buckenham et al. [29]	2008	4	IA-S	NE (1:1)	- Endoleak resolution 100%; - Two patients had minor increase in sac size (50%); - No complications.	359 (173–365) days
Chao et al. [30]	2006	4	TL-nS (3) IA-nS (1) TA-nS (2)	NE (1:3) + coils	- Endoleak resolution in 3 patients (75%); - Reintervention in 1 patient (25%; 2 repeats); - No complications.	55.5 (30–78) months
Choi et al. [24] *	2010	2	TA-nS	NE (1:1–3) ±coils	- Technical success 100%; - Clinical success 100%; - No complications.	16.4 (0–37) months (whole cohort)
Nakai et al. [25] *	2015	7	IA-S (3) TL-nS (2) TA-nS (2)	NE	- Technical success 100%; - Endoleak resolution 100%.	30.4 (12–48) months (whole cohort)

Footnote: TL: translumbar; IA: intra-arterial; TC: transcaval; nS: non-selective; S: selective; NBCA: n-butyl cyanoacrylate; NE: mixture containing n-butyl cyanoacrylate and ethiodized oil; ENOH: ethynele-vinyl alcohol copolymer. Follow-up was reported in mean ± standard deviation or median (range), if not otherwise stated. *: Study sample consisted of mixed populations, but data on patients treated with NBCA embolization for type II endoleak were extractable.

**Table 2:** Comparative studies reporting on NBCA-treated patients for type II endoleak.

Author	Year	Comparison Groups	Technical Aspects	Main Findings	Follow-Up
Fujitsuna et al. [31]	2024	- NE: 15; - NEE: 9.	- IA-S or direct sac puncture-nS (TA or TL); - NE: 1:2 to 1:5; - NEE: 1:5:0.5.	- Freedom from sac enlargement at 1 year 65% (NE) vs. 87.5% (NEE; *p* = 0.03); - Freedom from reintervention at 1 year 69.2% (NE) vs. 100% (NEE; *p* = 0.02); - No adverse events.	NE: 960 d (±1007) NEE: 484 d (±192)
Stavropoulos et al. [32]	2009	- IA (coils): 23; - TL (coils + NE): 62.	- IA-S: coils; - TL-nS: coils + NE (1:2 ratio).	- Technical success 96% (IA) vs. 100% (TL); - Clinical success 78.3% (IA) vs. 72.6% (TL; *p* = 0.41); - Major complications 0% (IA) vs. 3.2% (TL).	18.7 m (6–84)
Ushijima et al. [33]	2020	- 16 patients; - DP: TL or TA (10 sessions); - IA: 11 (sessions).	- DP-nS: NE (1:1 to 1:4); - IA-S: NE (1:1 to 1:4)± coils.	- Procedure time: 100 ± 35 (DP) vs. 191 ± 77 (IA; *p* < 0.05); - Contrast: 12.8 ± 7.1 (DP) vs. 71.8 ± 36.1 (IA; *p* < 0.05); - Technical success: 91% (DP) vs. 87% (IA). Complications: - DP: 1 arterial injury and 1 migration of NE in aortic lumen; - IA: 1 arterial injury.	-

Footnote: NBCA: n-butyl cyanoacrylate; NE: mixture containing n-butyl cyanoacrylate and ethiodized oil; NEE: mixture containing n-butyl cyanoacrylate, ethiodized oil and ethanol; IA: intra-arterial; TL: translumbar; DP: direct puncture; TA: transabdominal; S: selective; nS: non-selective; vs.: versus; d: days; m: months; Follow-up time is expressed as mean (± standard deviation) or median (range).

**Table 4:** Technical studies reporting on patients with type II endoleak managed with NBCA embolization.

Author	Year	Clinical Context	Access Route	NBCA Mixture	Follow-Up	Main Findings—Recommendations
Shimohira et al. [47] *	2013	Type II endoleak after EVAR (1 case)	IA-S (1)	NE (17% NBCA)	4 months	Triaxial catheter system - Smaller endoleak; - Stable size.
Saguchi et al. [48]	2024	Type II endoleak after EVAR (3 cases)	IA-S	NE (20% NBCA)	-	Quintet-coaxial catheter system - No complications; - Endoleak resolution.
Okada et al. [49]	2024	Type II endoleak after EVAR (1 case)	IA-S	NE (1:4)	5 months	Triple coaxial microcatheter system through deep iliac circumflex artery - No complications; - Endoleak resolution.
Hongo et al. [50]	2014	Type II endoleak after EVAR (20 cases)	IA-nS	NE (20–30% NBCA)	18.5 (6–36) months	Double coaxial microcatheter system - Technical success 90%; - Clinical success 80%; - Two patients (10%) developed back pain.
Mine et al. [51] *	2020	Type II endoleak after EVAR (2 cases)	IA-S	NE (1:3)	4.5 (1–10) months	Triaxial microcatheter system for transarterial warmed NBCA embolization under balloon occlusion - Technical and clinical success in both cases.
Katada et al. [52]	2013	Type II endoleak after TEVAR/EVAR (2 cases)	DP-nS	NE (1:1–2)	4.5 (3–6) months	Coaxial DP technique - No complication; - No endoleak; - No sac increase.
Kamada et al. [53]	2025	Persistent type II endoleak after TEVAR (3 cases)	IA-S (2) IA-nS (1)	NE (1:2)	4 (1–6)	Use of flow-directed microcatheters - Endoleak type II in 1/3 patients (33% in follow-up); - No aneurysm enlargement.
Ogawa et al. [54]	2019	Type II endoleak after EVAR (6 cases)	IA-nS (1) IA-S (3) DP-S (1) DP-nS (1)	NE (1:2–3)	10 months	Balloon aortic occlusion - Complete embolization of all involved branches by 50%; - One adhesion of NBCA to the microcatheter.
Midulla et al. [55]	2012	Persistent type II endoleak after EVAR (1 case)	TC-nS	NE	-	Transcaval embolization using transseptal needle-sheath - No endoleak post-op; - No complication.
Ogawa et al. [56]	2015	Type II endoleak after EVAR (1 case)	TP-nS	NE (1:3)	8 months	Isocenter puncture DP technique - Endoleak resolution; - No sac increase.
Churchill et al. [57]	2021	Type II endoleak after EVAR (1 case)	TA-nS	NE (1:3)	2 months	Contrast-enhanced ultrasonography - Endoleak resolution; - Stable size.

Footnote: IA: intra-arterial; DP: direct puncture; TC: transcaval; TP: transpendicular; TA: transabdominal; NBCA: n-butyl cyanoacrylate; NE: mixture containing n-butyl cyanoacrylate and ethiodized oil. Follow-up is reported in mean ± standard deviation or median (range). Follow-up was reported in mean ± standard deviation or median (range). *: Study sample consisted of mixed populations, but data on patients treated with NBCA embolization for type II endoleak were extractable.

**Table 5:** Case reports presenting complications after NBCA embolization for the management of type II endoleak after EVAR.

Author	Year	Technical Approach	Mixture	Complication	Management
***Embolic***					
Forester et al. [58]	2002	Intra-arterial, selective	NBCA– tantalum powder	Ischemic sciatic neuropathy, right sided foot drop	Conservative management, limited recovery
Sanz- Sánchez et al. [59]	2019	Intra-arterial, selective	NE (1:5 ratio)	Spinal cord ischemia leading to paraplegia	Methylprednisone injection and CSF drainage (only minimal improvement with minimal hip and knee flexion)
Gabel et al. [60]	2018	Translumbar, non-selective	NBCA (no info)	Ischemic colitis (sigmoid colon)	Sigmoidectomy
Saiga et al. [61]	2022	Intra-arterial, selective and non-selective	NE (5% NBCA for selective +10% for non)	Ischemic lumbar plexopathy, sensory/motor impairment of anteromedial inner thigh	Rehabilitation (persisted, not solved)
Chun et al. [14]	2020	Intra-arterial, non-selective	NE (1:4 ratio)	Spinal cord ischemia and ischemic radiculopathy (pain)	No management, gradual symptom elimination
Metias et al. [62]	2015	Translumbar, non-selective	NE (1:4 ratio)	Acute limb ischemia due to distal glue embolization	Arteriotomy and selective embolectomies
Lanza et al. [63]	2014	Intra-arterial, selective	NE (1:2)	Colonic ischemia, peritoneal fistula	Total colectomy, iliorectal anastomosis, protective ileostomy
Ito et al. [64]	2012	Intra-arterial, selective	NE (1:4)	Ischemic colitis	Conservative management
Bailey et al. [65]	2011	Intra-arterial, non-selective	NE (3.5:1)	Ischemic skin ulceration	Conservative, self-limiting
***Other***					
Ueda et al. [66]	2026	Translumbar, non-selective	NEE (1:4:1)	Acute bilateral lower limb ischemia from compression	Kissing stents, thromboaspiration
Maleux et al. [67]	2026	Translumbar, non-selective	NE (1:2)	Limp graft occlusion due to external compression	No treatment (asymptomatic)
Ni et al. [68]	2018	Translumbar, non-selective	NE (10% NBCA)	Ovation stent graft extrinsic compression of the unsupported part	Kissing balloon angioplasty (stenosis resolved)
Al Adas et al. [13]	2018	Translumbar, non-selective (transarterial selective failed)	NE (1:5 ratio)	Hemodynamically significant compression of the right iliac graft limb (intra-OP)	Balloon angioplasty (stenosis resolved)
Robinson et al. [69]	2024	Transcaval, non-selective	NBCA (no info) + coils	Graft infection	Endograft explantation and implantation of cryopreserved allograft
Balézeaux et al. [70]	2024	Translumbar, non-selective	NE (1:4)	Aortic infection	Retroperitoneal exploration, aortoiliac bypass.
Li et al. [71]	2019	Transabdominal, non-selective	NE (no ratio reported)	Graft infection	Endograft explantation and neoaortic reconstruction

Footnote: NBCA: n-butyl cyanoacrylate; NE: mixture containing n-butyl cyanoacrylate and ethiodized oil; NEE: mixture containing n-butyl cyanoacrylate, ethiodized oil and ethanol; CSF: Cerebrospinal Fluid.

**Table 6:** Studies reporting on pre-emptive embolization with NBCA for the preventive management of endoleaks type II.

Author	Year	Study Period	Patients	Mixture	Important Reported Outcomes	Follow-Up
Okada et al. [73]	2024	2018–2019	12	- NE: 40–60% NBCA selective; - NE: 20–25% NBCA non-selective.	- AAA diameter reduction rate (3.2%); - Residual TII endoleak after 6 months (2/12; 17%); - No complications.	6 months
Nakai et al. [74]	2016	2013–2015	24	NEE: 1:5:0.5 (non-selective)	- SPI 0.36 and 0.57, with and without proximal neck occlusion; - No complications; - No aneurysm increased in diameter; - Minor TII endoleak at follow-up (3/24; 12.5%).	12.1 months ± 5.8
Miura et al. [75]	2020	2013–2018	- 83 (NBCA group); - 93 (control).	NE: 1:6 to 1:9 (non-selective)	- TII endoleak at 7 days 2.4% vs. 22.6% (*p* < 0.001) - AAA diameter reduction at 12 months 6.6 mm vs. 4.4 mm (*p* = 0.026); - No complications.	12 months
Miura et al. [76]	2024	2013–2020	- 106 (NBCA group); - 81 (control).	NE: 1:6 to 1:9 (non-selective)	- TII endoleak at 7 days 2.8% vs. 28.4% (*p* < 0.001); - AAA diameter reduction at 12 months 5.2 mm vs. 3.8 mm (*p* = 0.034); - 2 cases of transient lower-limb motor dysfunction; - Survival without AAA enlargement (100% vs. 69.8%, *p* = 0.014); - Survival without reintervention (100% vs. 63.1%, *p* = 0.034).	12 months

Footnote: NBCA: n-butyl cyanoacrylate; NE: mixture containing n-butyl cyanoacrylate and ethiodized oil; NEE: mixture containing n-butyl cyanoacrylate, ethiodized oil and ethanol; AAA: abdominal aortic aneurysm; TII: type II (endoleak); SPI: systolic pressure index; follow-up time is expressed as absolute number or mean ± standard deviation.

## Data Availability

Data are available upon request.

## Supplementary Materials

The following supporting information can be downloaded at: https://www.mdpi.com/article/10.3390/jcm15218596/s1, Table S1: The PCC framework of the scoping review; Table S2: Search strategy of the scoping review.
