# Supplementary material for: N-Butyl Cyanoacrylate Use for the Management of Type II Endoleak in Endovascular Aortic Repair: A Scoping Review
Source: J Clin Med. 2026 Nov 4;15(21):8596. doi: 10.3390/jcm15218596 (PMC13763598; doi:10.3390/jcm15218596)
Supplement: Supplementary file 1 [file jcm-15-08596-s001.zip › jcm-4549481-supplementary.pdf]

P	Population	Patients facing type II endoleaks in the frame of endovascular aortic repair
C	Concept	Use of N-butyl-cyanoacrylate (NBCA) as an endovascular embolic agent for the management of type II endoleaks
C	Context	Endovascular or interventional radiology settings
	What type of question is asked?	What are the efficacy, safety outcomes and technical considerations of the use of NBCA for the management of type II endoleaks in patients undergoing endovascular aortic repair.

Supplementary table S1. The PCC framework of the scoping review.

<table>
<tr><th>Frame</th><th>Search terms (used both as full text and “MeSH” terms, when available)</th><th>Search</th><th>Inclusion criteria</th><th>Exclusion criteria</th><th>Sources</th></tr>
<tr><td>P (population)</td><td>#1 “endoleak”,
#2 “aneurysm sac perfusion”</td><td rowspan="4">#1 OR #2 AND #3 OR #4 OR #5 OR #6 OR #7</td><td rowspan="4">Any type of primary study including observational (retrospective or prospective), trials (randomized or non-randomized) case reports, technical notes reporting on NBCA use for the management of type II endoleak in patients undergoing endovascular aortic repair.
Secondary studies (any type of review)
In vitro studies
Animal studies
Peer reviewed journals
English language</td><td rowspan="4">Irrelevant title
Irrelevant full text
Non-English
Not applicable study type (editorial, comments)
Studies reporting on other types of endoleaks.
Studies reporting on other types of liquid embolics
Studies with non-extractable data for NBCA embolic</td><td rowspan="4">PubMed, Cochrane library, Scopus</td></tr>
<tr><td>C (Concept)</td><td>#1 “NBCA”,
#2 “n-butyl-cyanoacrylate”,
#3 “cyanoacrylate”,
#4 “Glubran”,
#5 “Histoacryl”,
#6 “Trufill”,
#7“glue”</td></tr>
<tr><td>C (Context)</td><td>NA</td></tr>
<tr><td>Time</td><td>Last search: 25.06.2026</td></tr>
</table>

Supplementary table S2. Search strategy of the scoping review.
